# Fabrication of Acrylonitrile-Butadiene-Styrene Nanostructures with Anodic Alumina Oxide Templates, Characterization and Biofilm Development Test for *Staphylococcus epidermidis*

**DOI:** 10.1371/journal.pone.0135632

**Published:** 2015-08-18

**Authors:** Camille Desrousseaux, Régis Cueff, Claire Aumeran, Ghislain Garrait, Bénédicte Mailhot-Jensen, Ousmane Traoré, Valérie Sautou

**Affiliations:** 1 Clermont Université, Université d’Auvergne, C-BIOSENSS, EA 4676, BP 10448, F-63000 Clermont-Ferrand, France; 2 Clermont Université, Université Blaise Pascal et Université d’Auvergne, LMGE, UMR CNRS 6023, F-63000 Clermont-Ferrand, France; 3 CHU Clermont-Ferrand, Service d’Hygiène Hospitalière, F-63003 Clermont-Ferrand, France; 4 Clermont Université, Université d’Auvergne, CIDAM, EA 4678, BP 10448, F-63000 Clermont-Ferrand, France; 5 CHU Clermont-Ferrand, Service Pharmacie, F-63003 Clermont-Ferrand, France; Université de Technologie de Compiègne, FRANCE

## Abstract

Medical devices can be contaminated by microbial biofilm which causes nosocomial infections. One of the strategies for the prevention of such microbial adhesion is to modify the biomaterials by creating micro or nanofeatures on their surface. This study aimed (1) to nanostructure acrylonitrile-butadiene-styrene (ABS), a polymer composing connectors in perfusion devices, using Anodic Alumina Oxide templates, and to control the reproducibility of this process; (2) to characterize the physico-chemical properties of the nanostructured surfaces such as wettability using captive-bubble contact angle measurement technique; (3) to test the impact of nanostructures on *Staphylococcus epidermidis* biofilm development. Fabrication of Anodic Alumina Oxide molds was realized by double anodization in oxalic acid. This process was reproducible. The obtained molds present hexagonally arranged 50 nm diameter pores, with a 100 nm interpore distance and a length of 100 nm. Acrylonitrile-butadiene-styrene nanostructures were successfully prepared using a polymer solution and two melt wetting methods. For all methods, the nanopicots were obtained but inside each sample their length was different. One method was selected essentially for industrial purposes and for better reproducibility results. The flat ABS surface presents a slightly hydrophilic character, which remains roughly unchanged after nanostructuration, the increasing apparent wettability observed in that case being explained by roughness effects. Also, the nanostructuration of the polymer surface does not induce any significant effect on *Staphylococcus epidermidis* adhesion.

## Introduction

Polymers are commonly used in perfusion medical devices including vascular catheters or their connectors. Vascular catheter-related infections are a public health concern [1]. The role of biofilms in these infections is clearly established. To avoid infections, different material strategies have been tested, for example by addition of biocidal substances, with the risk of favoring bacterial resistance, or by physico-chemical surface properties modifications, in order to limit bacterial adhesion. Bacterial adhesion is a multifactorial phenomenon: the properties of the surface material and those of the bacteria and the environment where the adhesion takes place are all important factors that can interfere with adhesion [2]. The adhesion depends on chemical surface properties of bacteria such as hydrophobicity and surface charge but also on the chemistry and topography of the materials. It is generally accepted that hydrophobic cells attach more strongly to a surface and that all bacteria tend to adhere more strongly to hydrophobic material [3,4]. The physical characteristics of the material surfaces have long been neglected in the bacterial adhesion theories. Some topographic or nanoroughness modifications of the surfaces can change their properties, such as apparent wettability [5–8]. Furthermore, nanostructured modified surfaces could interact mechanically with bacterial rigid cell wall and bacterial structures such as pili, giving them a stronger point of attachment [6,9]. The up-until now admitted fact that smooth surfaces are optimal to avoid bacterial adhesion is questioned [10,11]. A few engineered surfaces with micrometric or sub-micrometric topographical features have succeeded in reducing bacterial adhesion [12–14]. Currently, the reasons why a micro or nanofeature (size, density, shape etc.) is efficient or not against bacterial adhesion are not well understood. Therefore, strategies based on modifications of the surface chemistry or topography could be of interest to limit bacterial adhesion and subsequent infections.

Several methods to nanostructure surfaces have been described in the literature [6]. For polymers, fabrication processes can be divided into two categories. First, there are the templateless processes. Specific properties of coblock copolymers or conductive polymers and polymer demixing may lead to formation of nanofeatures [15–18]. Polymers can also be nanostructured by plasma or laser treatment [19–21]. Secondly, there are the replication processes involving a nanostructured mold. The final application of the nanostructures is a polymer piece of a medical device made by injection, that is why nanostructuration methods via a mold are selected. Molds are generally in metal (stainless steel) or in silicon. They can be nanostructured by many techniques (lithography, femtolaser, e-beam radiation, chemical etching, anodization…). Processes to nanostructure the mold by lithography are highly expensive. Currently, Anodic Alumina Oxide (AAO) is a template which can overcome this drawback. It is characterized by a periodical arrangement of hexagonal nanopores. It is generally produced by electrochemical anodization. AAO templates are used in this study because of their advantages: cheap fabrication, autonomous laboratory fabrication, and a wide range of possible nanopore dimensions [22]. AAO template is widely used as a mold to make nanopillars or nanotubes in polymers. Wetting or melting techniques are frequently used for polymers to replicate nanofeatures of AAO molds [23–26]. Infiltration of polymer fluids (melt or in solution) in nanopores can be a spontaneous process or be forced by application of external force as vacuum for instance [27,28].

The specific aims of this work were: firstly, to check the reproducibility of the AAO mold fabrication process, then to study the replication of the mold with thermoplastic acrylonitrile-butadiene-styrene ABS using different methods based on wetting and melting techniques and to select and optimize one method in regards with industrial application and reproducibility aspect, which is not often studied in other studies. Secondly, the selected nanostructured surfaces were characterized. Finally, the impact of nanostructures on bacterial adhesion and biofilm development of *Staphylococcus epidermis*, which is one of the most frequently involved germs in catheter biofilms, is investigated.

## Material and Methods

### Anodic Alumina Oxide templates Fabrication

AAO templates were made via a two-step anodization method of aluminum sheets (99.999%, Goodfellow). The anode was made of aluminum (Al) and the cathode of platinium. Before anodization, the Al foil was ultrasonically cleaned successively in trichloroethylene, acetone, methanol, deionized water and then degreased in a mixed solution of acids (HF/HNO_3_/HCl/H_2_O 1:10:20:69 in volume) and rinsed with deionized water. Then the cleaned aluminum sheets were electrochemically polished in a solution of perchloric acid mixed with ethanol (1:3 in volume) under a constant DC voltage of 15 V for 5 min. The sheets were then anodized at 40 V in 0.3 M oxalic acid electrolyte at 3°C for 17 h. After this first anodization, the aluminum oxide layer was dissolved in a mixed solution of 6 wt% phosphoric acid and 1.8 wt% chromic acid (1:1 in volume) at 60°C for 3 h and washed with deionized water. In order to obtain the highly ordered pores, the aluminum was anodized again under the same conditions as the first step. The obtained pore length is proportional to the anodization time: lengths of the mold pore of 100 nm correspond to second anodization time of 2 min 30. Pore diameters can be increased by chemical etching in 0.3 M oxalic acid solution at 30°C: pores of 55 nm correspond to 3 hours of etching.

Reproducibility of AAO nanopores was investigated using a Zeiss Supra 55 VP scanning electron microscope (SEM) after a gold sputtering. Three random areas were observed for each AAO sheet. For each area ten diameters and ten interpore distances were measured with GIMP software (version 2.8.0). In order to observe more easily the pore depth, AAO sheets were scratched with a scalpel. For each area, at least 5 pore lengths were measured.

### Polymer nanostructuration

Three techniques were used to nanostructure the ABS polymer (Novodur HD203FC, Styrolution).

#### Technique 1: Polymer Solution Wetting Method

ABS was dissolved in chlorobenzene to prepare a 10 wt% ABS solution whose viscosity was controlled with a Brookfield RDV-II Viscosimeter. 200 μL of ABS solution were poured onto the AAO templates (5x5 mm^2^). The solvent was allowed to evaporate overnight. The AAO/ABS complex was removed from the well then the ABS sample was removed from the AAO mold.

#### Polymer Melt Wetting Method

a. Technique 2: Polymer film. Drops of ABS solution were placed on a microscope slide. The solvent was allowed to evaporate overnight. The ABS films were then removed from the slide and placed for 10 minutes onto 175°C heated AAO molds placed on a hot plate. Then, the hot plate was allowed to slowly cool to room temperature and the ABS/AAO complex was removed from the hot plate and the ABS sample was peeled off from the AAO mold.

b. Technique 3: Melting of an ABS preinjected sample. ABS samples (4x1x0.1 cm^3^) were obtained by the injection molding of ABS with a Protoject KAP injection molding machine at 240°C. Then, 1x1 cm^2^ ABS was placed on the AAO mold heated at 135°C on a hot plate for 10 min. A 100 g weight was put on the polymer. After cooling of the hot plate, ABS/AAO complex were gently separated.

### Characterization of nanostructured polymers

Nanostructured and control surfaces were characterized. For each technique, three specimens of each sample were tested.

#### Topographical characterization

The surface topography characterization of the polymer surfaces was performed using a Zeiss Supra 55 VP scanning electron microscope (SEM) with secondary electron and in-lens detectors. Polymers were sputtered with carbon before observation. SEM images were used for a reproducibility study. This study controlled interpillar distances which is measured from center to center of the top extremity of the nanopillars, as well as nanopillar diameters and heights. For each sample, three areas were analyzed and 5 to 10 measures by area were taken for each dimensional parameter. For each parameter, the mean value, standard deviation and variation coefficient were calculated.

Innova Brucker atomic force microscope (AFM) was operated in tapping mode to complete the observation for the length parameter.

#### Polymer Characterization

a. Thermal analysis by DSC. Bulk ABS polymer, control and nanostructured surfaces were tested using a Perkin Elmer Instrument DSC 4000 under a constant nitrogen flow. The temperature cycle was composed of a first phase of heating from 30 to 200°C with a rate of 10°C/min, then cooled to 30°C with a rate of 40°C/min, followed by a stable phase at 30°C for 2 min and a second phase of heating from 30 to 200°C with a rate of 10°C/min.

b. Chemical analysis by IR spectrophotometry. ATR-infrared spectrophotometry was performed for chemical analysis of the top surface layer of the ABS using Perkin Elmer Spectrum 100 FTIR spectrophotometer. The ATR crystal was Diamond/Znse. For each sample, 20 scans with a resolution of 4 cm^-1^ and wavenumber ranging from 4000 to 650 cm^-1^ were collected.

#### Wettability study

a. Rough solid approach. The wetting behavior of an ideal flat homogeneous solid is assessed by the contact angle *θ* of a drop on its surface, given by the Young's relation:

$$\text{cos}\theta =\frac{\gamma_{SV}-\gamma_{SL}}{\gamma_{LV}},$$ (1)

where *γ*_*SV*_, *γ*_*SL*_ and *γ*_*LV*_ are the interfacial tensions of the solid/vapor, solid/liquid and liquid/vapor, respectively.

Wetting on non-ideal solid surfaces is much more complex since the contact angle of the drop can be dramatically affected if the solid is rough. The wettability of the solid thus depends on two factors, the surface chemistry and the surface roughness.

A simple model to characterize the influence of the surface roughness on the wettability of a solid was proposed by Wenzel [29]. In that model, this influence can be formulated by the prediction of the apparent contact angle θ* on a rough surface, considering the Young's contact angle on the flat surface *θ*, and the solid roughness *r* defined as the ratio between the real surface and the projected one:

$$\text{cos}\theta^{*}=r\text{cos}\theta$$ (2)

Experimental studies conducted by researchers of the Kao group have confirmed that the wetting properties were dramatically affected by surface roughness, but differently according to the wettability region [30,31].

In the hydrophilic domain θ* is found to be smaller than *θ*, while the contrary is observed in the hydrophobic side.

The experimental representation of cosθ* *vs* cos*θ* has shown that the evolution observed was not a simple linear relation, two consecutive linear regimes are expected whether the roughness is flooded or not.

For hydrophilic solids, the roughness makes the surface more wettable and the first regime can be described by the Wenzel relation (2). This Wenzel regime, which assumes a dry solid upon the contact line of the drop, is observed when *θ* exceeds the critical angle value *θ*_*c*_ which fixes the crossover between the two regimes [32].

*θ*_*c*_ is given by [8,33,34]:

$$\text{cos}\theta_{c}=\frac{\left(1-\phi_{S}\right)}{\left(r-\phi_{S}\right)}$$ (3)

where *ϕ*_*S*_ is the area fraction of the solid surface.

The second linear regime is settled as *θ* becomes smaller than *θ*_*c*_; it results from the penetration of the liquid inside the microtexture. Then, at odds with Wenzel hypothesis, the drop stands on a surface composed of liquid and solid [8,32]. The dependence of the apparent angle θ* with *θ* in the hydrophilic domain is then [32–34]:

$$\text{cos}\theta^{*}=1-\phi_{S}\left(1-\text{cos}\theta \right)$$ (4)

b. Contact angle measurements. The contact angle measurements on flat (control samples) and rough (nanostructured samples performed with technique 3) ABS surfaces were performed by using the captive bubble technique, with a contact angle measuring system (Krüss Drop Shape Analyzer DSA 14) involving angle measurements in a three-phase system consisting of water, solid surface and air. The measuring cuvet was filled with deionized water and polymer samples (surface of about 20mm^2^) were laid down on two PTFE sample holders placed in the cell. An angled deposition needle was carefully deposited bubbles (4μL) below the polymer sample. Then, the tilting angle of the camera was adjusted to identify the contact line between the captive bubble and the sample, and the DSA software calculated the contact angles with the solid surface.

The experimental contact angle values reported for the control (Young's contact angle *θ*) and nanostructured (experimental apparent contact angle $\theta_{\text{exp}}^{*}$) surfaces are the average of a minimum of 6 measurements made on 6 samples.

The theoretical predicted apparent contact angles θ* of the nanostructured polymer surface, which takes into account the geometry parameters of the structure (treated in the next paragraph) will be evaluated with eqs (2) or (4), depending on the regime observed (related to the Young's contact angle which is below or above the critical value *θ*_*c*_). As the nanostructuration of the polymer surface can dramatically affect its surface apparent wettability, the experimental and predicted apparent contact angles will be compared. A discussion on the respective effects of surface chemistry and surface roughness in the wetting evolution will be then proposed.

c. Surface roughness and topographical characterization. The nanopillars are arranged in hexagonal symmetry, thus, the area fraction of the solid surface, *ϕ*_*S*_, can be formulated as [35]:

$$\phi_{S}=\frac{\pi a^{2}}{2\sqrt{3}{\left(a+b\right)}^{2}}$$ (5)

where *a* is the diameter of the pillar and (*a+b*) the interpillar distance (center to center).

The surface roughness *r* for an hexagonally arranged structure of pillars of height h, can be expressed as [35]:

$$r=\frac{\sqrt{3}{\left(a+b\right)}^{2}+2\pi ah}{\sqrt{3}{\left(a+b\right)}^{2}}$$ (6)

### Biofilm formation assay

The bacterial strain used in this study was *S*. *epidermidis* CRBIP 21.25. The strain was stored at -80°C. Aliquots were thawed at 4°C then grown at 37°C in Trypton Soja Agar plates (TSA). Bacteria were suspended in a Trypton Soja Broth (TSB) buffer and the bacteria concentration was measured by a spectrophotometer at 620 nm.

For each microbiological experiment, control and nanostructured 5x5mm^2^ surfaces were studied. Samples were disinfected in ethanol before use. ABS samples were fixed with one drop of neutral glue (Eukitt, Fluka) on the bottom of 24 well plates to avoid swaying of ABS samples and were disinfected once again in ethanol. One mL of bacterial suspension (1.10^4^ CFU/mL inoculums) was added in each well. Incubation was made at 37°C whilst gently shaken for 3, 6, 24 and 48 h.

In order to determine the attachment of *S*. *epidermidis*, the medium was discarded with caution and the samples were gently rinsed with sterile physiological serum to remove non adherent bacteria. Then the samples were removed and placed in 5mL of Letheen broth. To recover the biofilm, the samples were vortexed for 5 min, sonicated for 5 min and vortexed again for 5 min. These solutions were serially diluted and viable plate counting was performed on TSA plate (n = 3).

### Statistical analysis

All parameters are given: mean ± Standard Deviation (Coefficient of Variation).

For measurement studies, means of dimensional parameters were compared using non parametric tests such as Mann-Whitney or Kruskal-Wallis test. For microbial experiments, concentrations of bacteria recovered from biofilm were analyzed using a Mann-Whitney test. For all tests, difference was considered significant at p<0.05.

## Results and Discussion

### AAO mold

**Fig 1:**
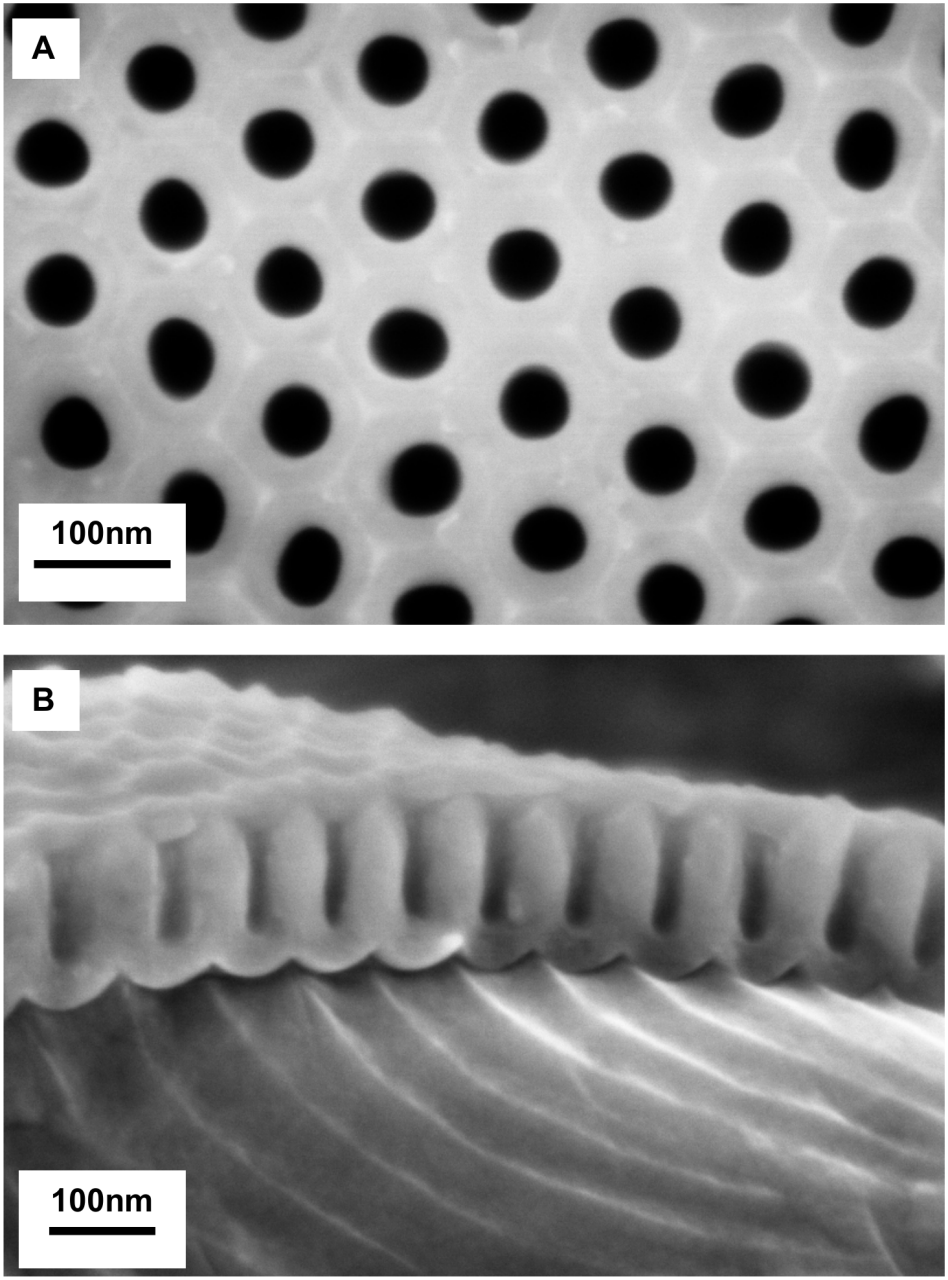
SEM images of AAO mold. (A) top view with 50 nm diameters and 100 nm interpore distances (B) cross-section view with pore lengths of about 100 nm

Highly ordered nanoporous AAO molds were obtained by double anodization in oxalic acid with a second anodization time of 2 min 30 and pore-opening for 3 h with cylindrical channels (Fig 1). The top view of AAO mold enables measures of pore diameters and distance between pores (Fig 1A). Hexagonally arranged cylindrical channels can be observed. The reproducibility study gives a mean interpore distance of 102 ± 6 nm (coefficient of variation CV: 6%, n = 90) which is in accordance with the distance obtained in other studies using oxalic acid at 40 V [36,37]. The mean diameter of the spherical pores is 51 ± 6 nm (CV: 11%, n = 90). Fig 1B shows a cross-section of the AAO mold. The mean length of the pore is 97 ± 9nm (CV: 9%, n = 45). The coefficients of variation are around 10%, which is satisfactory for our reproducibility study. We therefore assume that our protocol enables a reproducible fabrication of AAO mold with the following controlled parameters: diameters, lengths of pore and interpore distances. These parameters are rarely controlled in other studies on AAO although it is important to obtain reproducible molds in order to make reproducible nanostructured polymers. To assess the impact of nanostructures on biofilm adhesion, it is essential that they have no variability in their fabrication related characteristics.

### Polymer nanostructuration

**Fig 2:**
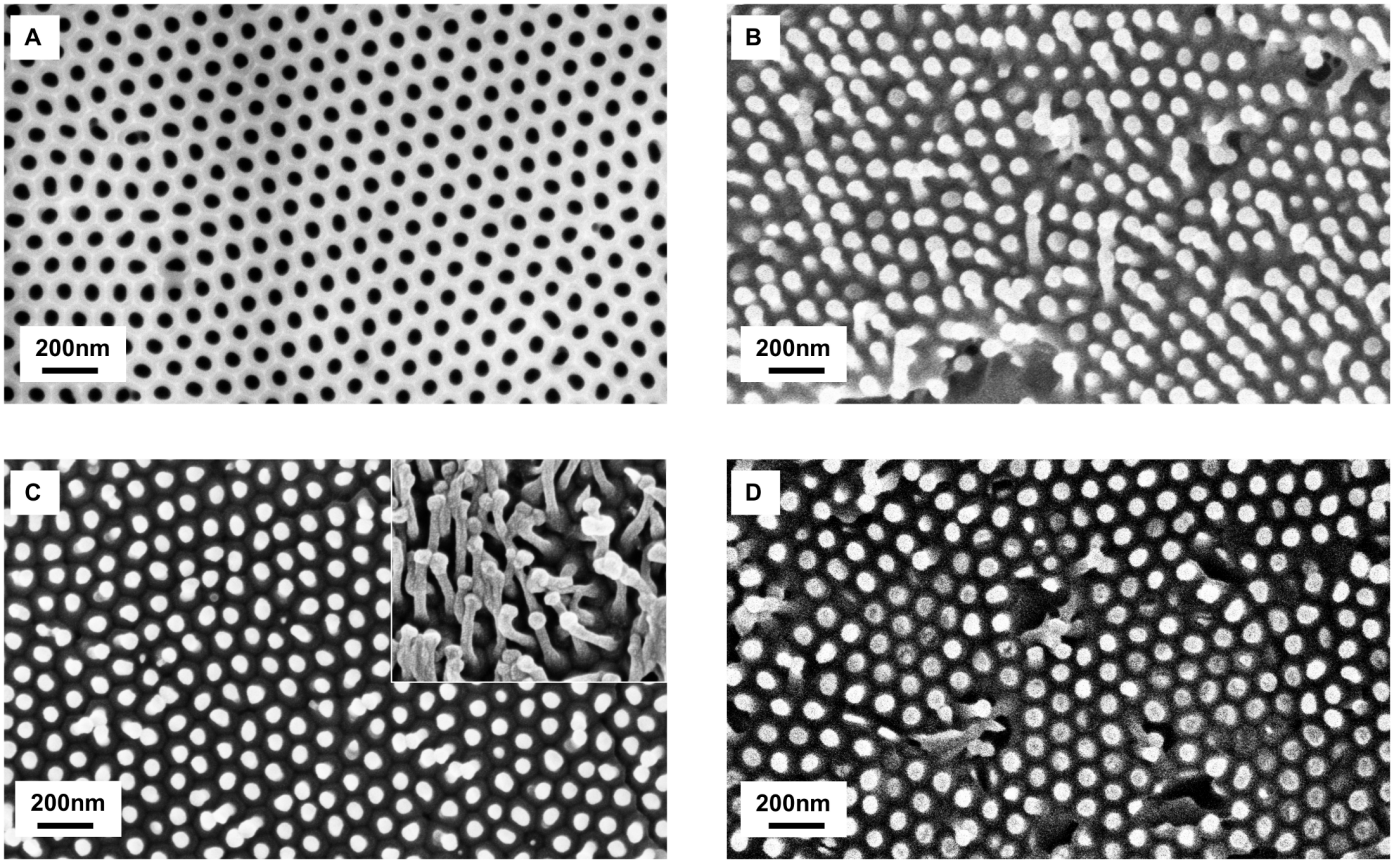
SEM images of AAO mold and nanostructured ABS. (A) AAO mold; (B) ABS nanostructured with polymer wetting solution (technique 1); (C) Two areas of nanostructured ABS Film (technique 2); (D) Injected ABS nanostructured with heat plate (technique 3)

Fig 2 shows SEM images of the nanostructured ABS surfaces replicated from AAO mold after demolding. The reproducibility study of the nanostructures dimensions for the three nanostructuration techniques are presented in Table 1.

**Table 1:** Results of the reproducibility study.

Techniques	Diameter (nm)		Interpillar Distance (nm)		Length (nm)	
	Measures	p	Measures	p	Measures	p
Technique 1: solution	61 ± 14 (22%)	<0.001	97 ± 11 (11%)	0.04	76 ± 23 (34%)	<0.001
Technique 2: film	51 ± 10 (20%)	0.64	100 ± 16 (16%)	<0.001	109 ± 53 (49%)	<0.001
Technique 3: preinjected ABS melting	56 ± 7 (13%)	0.80	101 ± 13 (13%)	0.34	73 ± 33 (45%)	<0.001

Each parameter is given: mean ±SD in nm (CV), n≥100 measurements.

The p-values are the results of Kruskal-Wallis tests comparing means of 3 or 4 samples.

#### Interpillar distances

ABS nanopillars are arranged in hexagonal symmetry and the interpillar distance is about 100 nm for the three techniques of fabrication, which is in consistent with the interpore distance of the AAO mold. However this parameter may vary if the nanopillars collapsed in bundles (Fig 2). Agglomeration of nanopillars could be explained by several factors: collapse due to self-weight, due to adhesion forces between the nanopillars and the base (ground adhesion) or due to lateral adhesion [23,38–41]. It has been shown that the lateral adhesion due to Van Der Waals interactions is the most significant factor [38,41]. Collapsing depends on the dimensions (aspect ratio: diameter/length) and the organization of the nanostructures (density, spacing, square array, hexagonal array) [38,39,41,42] and on the properties of the material, especially its stiffness and surface energy. For a same organization, materials with high stiffness can be used for high aspect ratio nanostructures without collapsing [39]. The Young modulus of the ABS tested in our study is of 1.4 GPa. This value is close to the modulus of poly(methyl methacrylate) PMMA (2.4–3.4 GPa). With an AAO mold, Goh *et al*. developed PMMA surfaces with nanopillars of 45 nm diameter, 130 nm length and 100 nm interpillar distance [42]. The hexagonal organization and 100 nm interpore distance was similar as our study. The aspect ratio of their nanostructures was around 3 and their PMMA nanostructures did not present bundles whereas with a 63 nm interpore distances, PMMA nanostructures collapsed [42]. In this work, aspect ratio is around 1.5–2 and there are a few bundles. This could be due to a slightly lower Young modulus of ABS compared to PMMA.

#### Diameters

The two melting techniques used produced nanopillars of about 55 nm in diameters, which is close to pores diameters of AAO. For the solution wetting technique, nanopillars diameters are wider than the diameters of the mold. With this last technique, the top extremity of the nanopillars seem to be swollen. To our knowledge, 200 nm diameter nanotubes were previously made with ABS material but there has been no report of ABS nanostructures with a sub-100 nm resolution [43]. It is known that when using stiffer materials a higher resolution can be achieved. For example, PMMA can molded into structures with a below 100 nm resolution [42,44]. However, nanostructures with sub-100 nm resolutions have also been made with less stiff materials like polystyrene PS, polypropylene PP, polyethylene PE, poly(vinyl chloride) PVC, polyurethane acrylate PUA or hard polydimethylsiloxane PDMS [23,27,35,36,45,46].

#### Lengths

According to the dimensions of our AAO mold, the nanostructures should have an aspect ratio inferior or equal to 2, which should enable a good replication. Indeed, with higher aspect ratios, difficulties have been reported (collapsing, incomplete filling, complex demolding) for injection processes [35]. In these cases, additional treatments are developed (control heating of the mold, coating of the mold) [23,45,46]. For the solution wetting technique (technique 1) and the melting technique with injected polymer (technique 3), the mean length of nanopillars are one quarter smaller than the length of nanopore. We hypothesized that the incomplete filling is due to air being trapped at the bottom of the pores [42]. For the film technique (technique 2), the mean length is about 100nm, but some nanopillars are longer than the depth of the mold. Such a finding could be explained by features stretching when the polymer is removed from the mold. Coefficients of variations, calculated with at least 3 samples, are more important for this parameter (between 34 and 49%) than for diameter and interpillar distance. This variation takes into account sample-to-sample variation, pillar to pillar variation for the same sample and the measuring error. The Table 2 presents the coefficients of variation for each of the 4 ABS preinjected samples made with technique 3 which are between 27 and 42%, which shows that there is a length variation intra-sample too. AFM microscopy was made on nanostructured samples made with the technique 3 in order to confirm the observations made for the lengths of the nanopillars. These observations confirmed the tendency that nanopillars present a variation for the lengths on the same sample and the height of the pillars are in the same order of magnitude. Results are 62 ± 26nm (coefficient of variation of 42%, for n>100 measures).

**Table 2:** Details of length data for nanostructured ABS with technique 3.

ABS sample	Length (mean ± SD) in nm	Coefficient of variation	Number of measures
ABS n°1	100 ± 32	31%	40
ABS n°2	76 ± 32	42%	30
ABS n°3	47 ± 13	27%	21
ABS n°4	68 ± 22	32%	30

### Technique selection

Reproducibility was one criterion to select the method of fabrication. Results of statistical tests show that the first and second techniques were not reproducible for each dimensional parameter whereas for the third technique two parameters (diameters and interpillar distances) are reproducible. Overall, preinjected ABS melting seemed to be the best method.

We believe that, in an industrial application aim, parameters other than the reproducibility should also be taken into account. Other selection criteria of a nanostructuration technique should include the ease and convenience for an industrial transposition, as well as environmental issues. Even if with solution wetting infiltrations, parameters such as the concentration or the quality of solvent can be controlled, melting solutions are usually more reproducible and solvent related problems, such as incomplete evaporation, are eliminated [27]. From an industrial transposition point of view, the use of a solvated polymer liquid solution is quite different from the current industrial process using an injection press with ABS pellets. The third technique is closer to the current injection process since there are no solvent in the ABS and the infiltration is only due to heating, whose temperatures can be reached for the mold during the injection process. Finally, minimizations of health and environment risks are also important parameters. Therefore, techniques using solvents were dismissed and additional work was focused on the optimization of the melting technique with pieces of preinjected polymer (technique 3).

### Optimization of the selected replication technique

The optimization of the nanopillars length was made by trying to reduce its variability and in particular to improve the filling of the mold pores. The objective is to obtain similar nanopillars on the same sample in order to limit intra-sample length variability and furthermore to limit inter-sample variability, in order to reduce the risk of dispersive results in the microbial tests due to variable surfaces.

For each optimization test, diameters of the nanopillars and internanopillar distances were kept constant and were in agreement with the dimensions of the AAO mold.

#### Mold dissolution

Initially, demolding was preferred to mold dissolution in order to be able reuse the mold, which is more suitable for industrial applications because the molten plastic may adhere to the mold insert and stay stuck in the mold or cause stretching when the ABS is removed from the AAO mold. To check this hypothesis, AAO molds were dissolved by etching in a saturated HgCl_2_ solution to remove the aluminum and by etching in a 30 wt% NaOH solution to remove the alumina to avoid various mechanical issues related to mold release. Results after dissolution of the AAO mold showed nanopillars with a variability of length (66 ± 32 nm CV: 48% n = 50). These results are quite similar to the demolding ones (77± 22 nm CV: 30% n = 30) with a p = 0.70 with a Mann-Whitney test. Overall, it seems that demolding is not the cause of variability of lengths or shortened nanopillars.

#### Depth of pores

**Fig 3:**
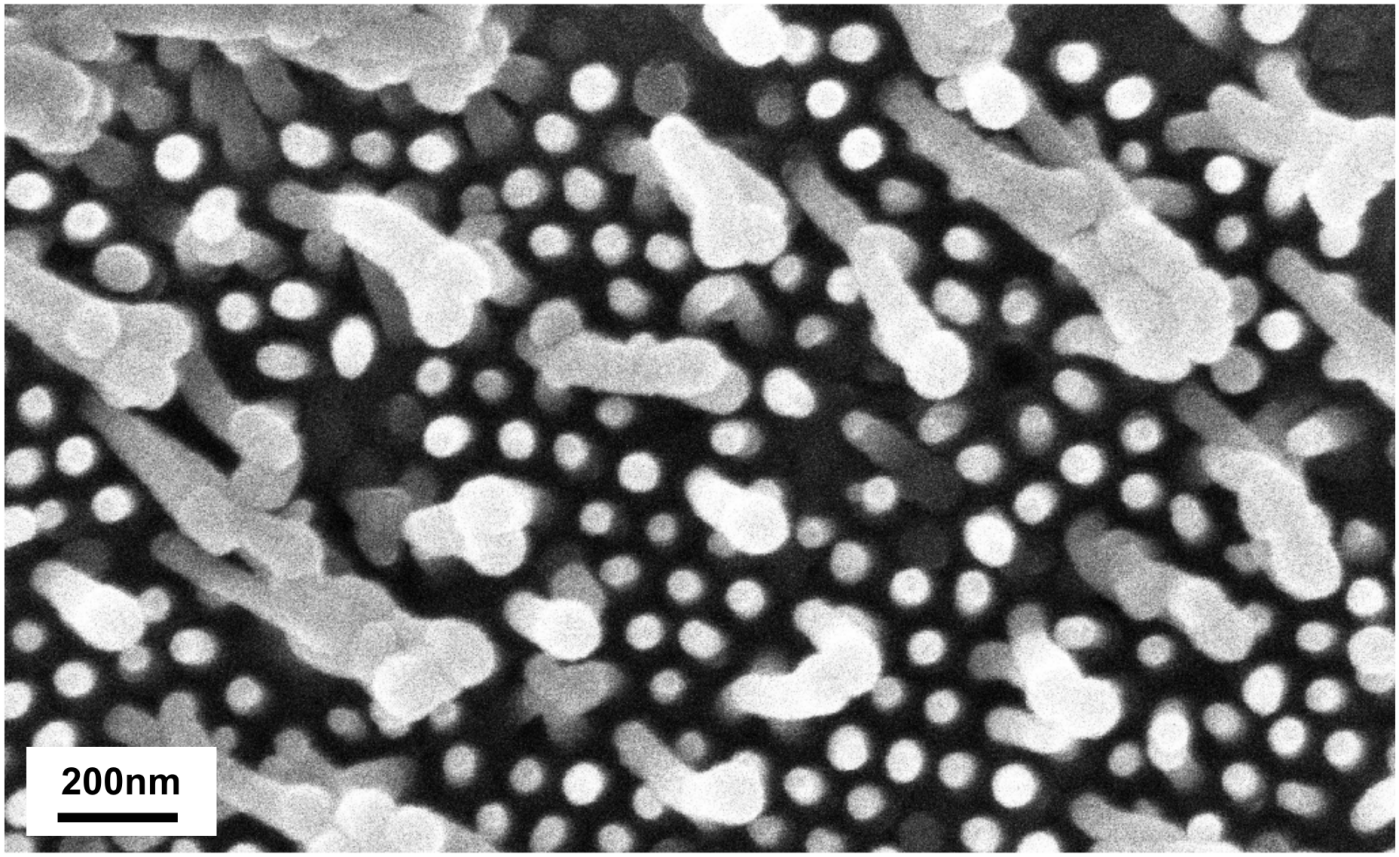
Stretching of nanopicots with a mold of 200nm-deep pores.

The relationship between filling and the depth of the nanopore was investigated. Different depths of nanopores were tested from 50 to 400 nm. Lengths of the mold pore of 50, 100, 200 and 400 nm correspond to second anodization times of 1 min 15, 2 min 30, 5 min and 10 min respectively. The ABS heating process was still of 135°C for 10 min (Table 3). The filling decreased with the depth of the pore, while the variability of the length increased. Fig 3 is representative of surfaces molded on AAO with longer pores, a majority of the pillars have almost the same length and a few are very much longer (stretching or a better filling). To reduce pillar to pillar variation inside the same sample, working with below or equal 100 nm deep nanopore seems preferable.

**Table 3:** Variation of nanopillars lengths according to AAO pore depths.

AAO pore depths (nm)	Nanopillar lengths (nm)	Filling rate (%)
50	65 ± 15 (23%)	131
100	72 ± 22 (30%)	72
200	153 ± 60 (40%)	77
400	170 ± 129 (76%)	43

Each parameter is given: mean ± SD (CV), n≥30 measurements

#### Duration of heating

The supposition that a prolonged heating process time was necessary to entirely fill the pore was assessed. Tests at 135°C for 10, 20 and 30 min were made. The analysis of the SEM images showed that the length of the pillars and their variability was similar (p = 0.29 with Kruskal-Wallis test) even with prolonged heating time (Table 4) [42]. Shorter times are favored for industrial production.

**Table 4:** Variation of nanopillar lengths according to duration of melting phase.

Duration of melting phase (min)	Nanopillar lengths (nm)	Filling rate (%)
10	72 ± 22 (31%)	72
20	78 ± 19 (24%)	78
30	70 ± 26 (37%)	80

Each parameter is given: mean ± SD (CV), n = 30 measurements

Optimization tests of the selected technique 3 did not enable a diminution of variability of the lengths. All the following nanostructured surfaces on this study (characterization and bacterial adhesion tests) are made with a heating of preinjected ABS at 135°C during 10min on a heated plate. Nanofeatures are hexagonally arranged nanopillars with dimensions of 73 nm length, 56 nm diameter and 100 nm interpillar distance

### Characterization

For DSC analysis, temperatures are based on the second heating step. The control and nanostructured surfaces presented no difference for DSC measurements (Table 5). The glass transition temperatures were around 105°C for all the samples, which corresponds to the ABS polymer signature temperature [47].

**Table 5:** Characterization of polymers: glass transition temperatures (DSC measurements) and contact angle values (water/solid/air system) of control and nanostructured ABS (technique 3).

ABS samples	Control	Nanostructured
Glass transition temperature Tg (°C)	104.9 ± 0.3	105.8 ± 0.6
Experimental contact angle (°)*	*θ* = 70.0±2.6	***$\theta_{\text{exp}}^{*}=43.6\pm 1.6\;$***

*θ is the angle for the flat control polymer and $\theta_{\text{exp}}^{*}$ is the experimental apparent contact angle for the nanostructured surface

Each parameter is given: mean ± SD, for Tg n = 3 measurements, for contact angle n = 6 measurements

**Fig 4:**
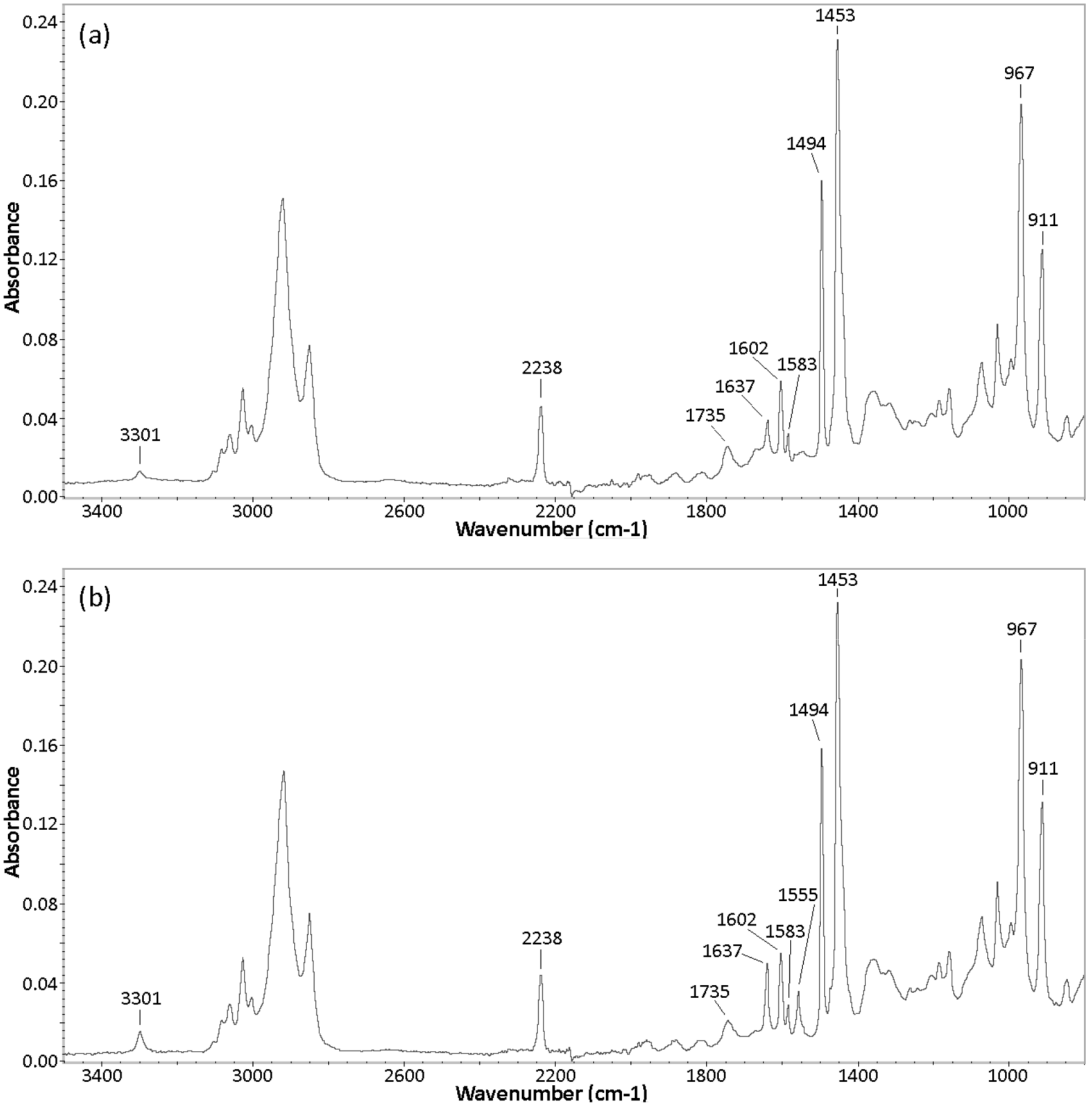
ATR-FTIR spectra of ABS samples: (a) Control; (b) Nanostructured with technique 3.

The ATR-FTIR spectra of the control and the nanostructurated ABS (Fig 4) were recorded to ensure that the nanostructuration process does not degrade the polymer. Many well-defined bands identified in both studied samples can be attributed to native ABS; amongst them:

- the aromatic and aliphatic C-H stretch modes are seen in the 3200–2800 cm^-1^ range,
- the CN stretching from acrylonitrile appears at 2238cm^-1^,
- the C = C stretching mode of poly(butadiene) is present at 1637cm^-1^,
- the aromatic ring in styrene is defined at 1602, 1583 and 1494 cm^-1^,
- the scissoring mode of the CH_2_ groups is located at 1453 cm^-1^,
- the C-H deformation for hydrogen atoms attached to alkenic carbons in poly(butadiene) is visible at 967 and 911 cm^-1^.

The peaks located at 3301 and 1735 cm^-1^, present in control and nanostructured ABS, are not consistent with native ABS. By comparing the two FTIR spectra, we can add that the absorbance of the peak at 3301 cm^-1^ is proportionally more pronounced in the nanostructured polymer than in the control ones. A similar observation is noticed for the peak at 1637 cm^-1^; which is clearly more intense in the nanotructured ABS than in the control polymer. The FTIR spectrum of the nanostructured polymer also shows the presence of an additional peak, appearing at 1555 cm^-1^.

Others authors have also reported the presence of 3 bands located at 3301, 1637 and 1552 cm^-1^ in commercial ABS [48]. They attributed the simultaneous appearance of these three peaks to a noncyclic N-monosubstituted amide, which may arise from a toughening and antistatic additive added to ABS by the manufacturer.

The presence of such an additive in our ABS sample is consistent with the FTIR spectra obtained. Indeed, the high absorbance of the peak located at 1637 cm^-1^ in the nanostructured polymer can result from an overlapping of the C = C stretch mode of ABS with the signal of the C = O stretch of the amide. Thus, the increasing intensity of the peaks at 3301 and 1637 cm^-1^ together with the appearance of the additional band at 1555 cm^-1^, supports the assumption that the amide is present at the surface of the nanostructured polymer. However, its presence in the control polymer cannot be ruled out, the results suggest that the nanostructuration process (heating) enhances the diffusion of the additive towards the ABS surface.

The second band which is not supposed to be present in native ABS, located around 1730 cm^-1^ in the two samples studied, is ascribed to contribution from carbonyl stretching of an ester group. It can be attributed to the presence of an additive in the ABS formulation, *i*.*e*. an hindered amine light stabilizer (HALS) [48] or a phenolic antioxidant [49,50].

The wettability experimental results, presented in Table 5 show that the flat control polymer surface presents a slightly hydrophilic character (*θ*<90°). The evolution of the contact angle indicates that the wetting effect is strengthened by nanostructuration; the experimental contact angle decreases from 70° on the flat surface to an apparent value of about 43° on the nanostructured polymer.

For the hexagonally arranged nanostructured ABS (performed with technique 3) a surface roughness value *r* of 2.48 and an area fraction of the solid surface *ϕ*_*S*_ of 0.28 were obtained with assuming a pillar diameter of 56 nm and an interpillar distance of 100 nm. The pillar height value, which presents a consequent variation, has been fixed at 73 nm, with respect to the average value reported in Table 3. These surface geometry parameters allow determination of the critical angle value *θ*_*c*_, which exhibits a value of 71°; then, the propagation of the liquid in the textured solid is governed by the second regime (*θ*<*θ*_*c*_) and the drop sits upon a mixture of solid and liquid.

Thus, the predicted apparent contact angles θ* of the nanostructured polymer surface has been evaluated with eq (4). By taking into account the large variation in surface geometry parameters, which prompts us to proceed with caution with the interpretation of the θ* contact angle value, the neighboring values obtained for the predicted apparent angle (36°) and the experimental ones (around 43°), strongly suggests that the major wetting effect involved by nanostructuration of ABS is mainly due to the roughness of the surface.

According to other works dealing with the nanostructuration of polymer surfaces with nanoporous AAO templates, it is not obvious to bring out a widely shared trend on the effect of nanostructuration on the wetting properties of the polymer surface.

On one hand, some authors noted an increase in the water contact angle values consecutive to nanostructuration of the polymer surfaces, as reported for polyolefin surfaces [51,52] or polystyrene substrate [36].

On the other hand, some studies revealed more nuanced aspects about the effect of nanostructuration on wettability. Miikkulainen *et al*. reported that wettability of PP surface is affected by the pore diameter of the nanostruture, the contact angle increased as the pore diameter decreased [35]. A similar evolution of the contact angle was observed for poly(*N*-isopropyl acrylamide) polymer [53]. According to the polymer studied, a temperature-dependent behavior may be noted. These authors added that the gradual decrease in the contact angles with increasing the pore size of the nanostructure substrate, when measured at ambient temperature, led on the opposite to a dramatic increase in contact angles when measurements occurred at a higher temperature (40°C). Other authors assert that surface nanostructuration has only a little effect on contact angle measurements (PDMS substrate), but when combined with chemical modification (plasma treatment), it can further improves the surface property, *i*.*e*. providing binding site for a coating [54].

As the effect of the surface texture strongly differs depending on the initial hydrophilic or hydrophobic character of the polymer, the consequences of an increasing surface roughness on the contact angle values will be necessarily various. This may somehow partly explains the different wetting effect observed on different nanostructured polymer substrates.

### Bacterial biofilm

**Fig 5:**
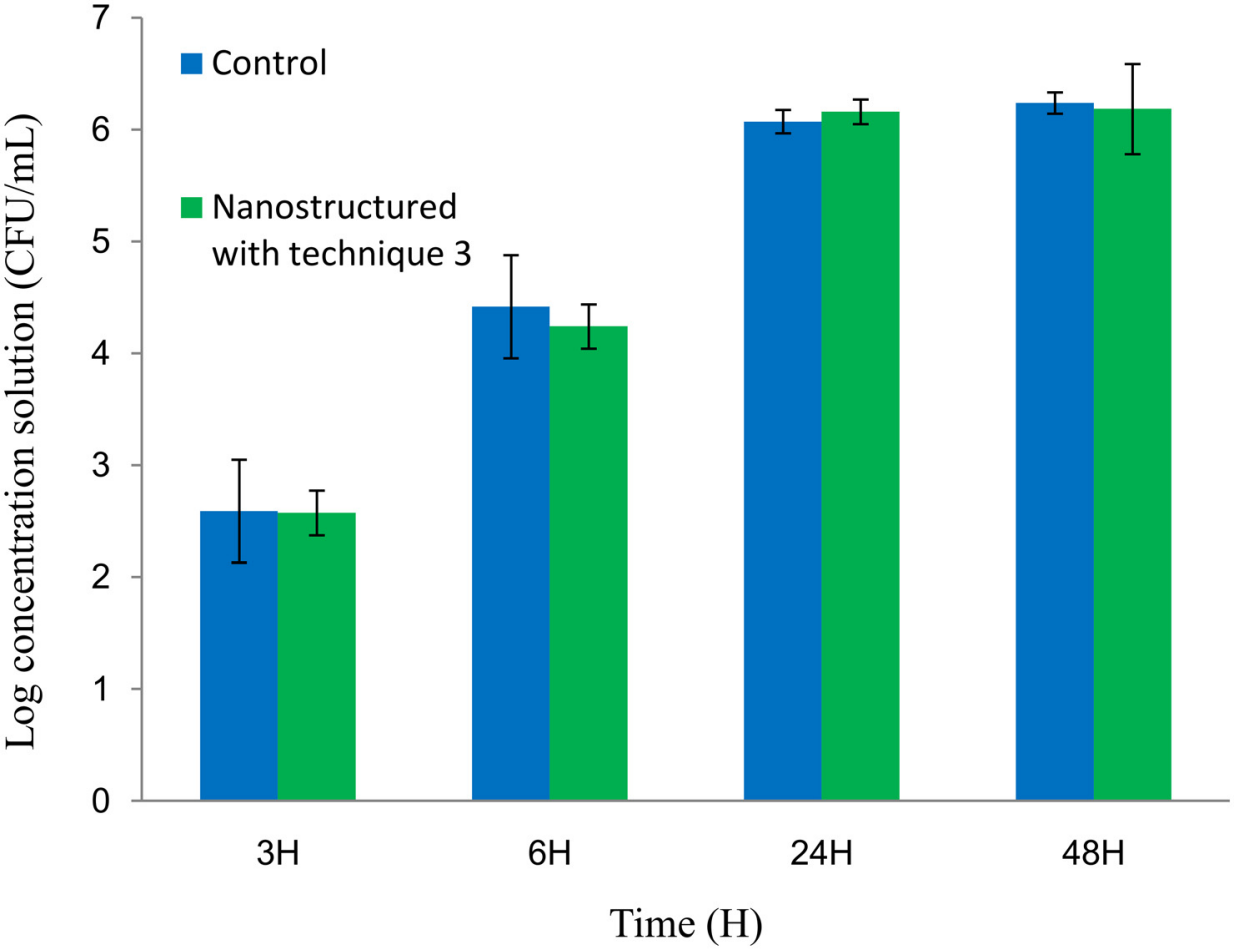
Concentration of viable *S*. *epidermidis* recovered from biofilm after different incubation times on control and nanostructured with technique 3 ABS samples.

Concentrations of bacteria recovered from biofilms were of about 300, 1.10^4^ and 1.10^6^ CFU/mL, equivalent to a bacterial density on samples about 60, 2.10^3^ and 2.10^5^ CFU/mm^2^, for incubation times of 3, 6 and 24/48h respectively (Fig 5). No difference was found between the control and the nanostructured surfaces (performed with technique 3) for each incubation time, corresponding to various levels of biofilm maturity (p values are 0.7, 0.4, 0.4 and 1 for 3h, 6h, 24h and 48h respectively). Several durations of bacterial incubation on the surfaces were used in the experiments in order to detect potential differences of adhesion between nanostructured and control surfaces according to biofilm formation steps (from adhesion to maturation). Indeed physical, chemical and biological interactions differ during the biofilm lifecycle, especially in relation with the extracellular matrix and the three-dimensional structure [55]. Furthermore, no difference in *S*. *epidermidis* CBRIP 21.25 adhesion between control surfaces and the ABS surfaces with these specific nanofeatures was noted for each incubation time.

We chose to use a culture method because it is a very classical reference method to quantify biofilms [56,57]. However this method has its drawbacks; especially it is important to control the conditions of bacterial recovery. Indeed, even if we sought to analyze only sessile bacteria, the removal of the medium and the rinsing step has to be done very cautiously in order not to disrupt the less adherent bacteria from the biofilm. The vortexing and sonication techniques applied to remove the biofilm from the surfaces in this study have been very frequently used for a large panel of medical devices [58,59]. With these treatments, most of the biofilm is removed and most importantly, with the aim of comparing several surfaces, the technique gives reproducible results as suggested by the low standard deviations. Finally the use of TSB culture medium could form a conditioning film on the surface making it difficult to conclude on the real impact of the surface modification on bacterial adhesion. However it is noteworthy that most of the real life clinical uses expose to a conditioning film with organic compounds such as blood [58,60].

As far as we know, there have been no previous studies about bacterial adhesion on surfaces similar to the one we developed (ABS with hexagonally arranged nanopillars with dimensions of 73nm length, 56 nm diameter and 100 nm interpillar distance).

As discussed in our recent literature review, it is very difficult to know whether a surface with specific nanofeatures will have an anti-adhesion effect [61]. Indeed, bacterial adhesion is a complex phenomenon where physico-chemical properties of the bacteria, of the substrate material and environmental conditions are all involved [6,61]. On a chemical level, the functionalities present at the surface of the material can impact the adhesion of bacteria. For example, Hook *et al*. showed that polymers with ester moieties (CHO_2_^-^) or cyclic hydrocarbon moieties (C_4_H^-^, C_6_H^-^) give less strong bacterial attachment than materials with ethyl glycol (C_2_H_3_O^-^, C_2_H_3_O^+^) and hydroxyl fragments groups (C_4_H_2_O_2_^-^, C_6_H_11_O_3_^-^). These results could not have been predicted with the current theories of bacterial adhesion [62]. In this work, the contact angles study seems to invalid the potential hypothesis that the nanostructuration process could significantly modify the chemical functionalities at the surface of ABS. Indeed, the wettability evolution suggests that the change in surface apparent wettability is mainly due to roughness effect and that the nanostructuration process does not induce any relevant chemical modification on the polymer surface. The slight hydrophilicity of the flat control polymer remains roughly unchanged after nanostructuration. It is admitted that bacterial adhesion tends to be reduced on hydrophilic material. Indeed, Self Autoassembled Monolayers functionalized with hydrophilic moieties (OH, NH_2_) tend to reduce bacterial adhesion compared to hydrophobic surfaces functionalized with methylated groups (CH_3_) [63]. However, a reduction of bacterial adhesion has been described in certain conditions both with superhydrophobic (angle > 150°) and superhydrophilic (angle < 5°) surfaces [64]. In our study, the absence of surface chemistry modification of the polymer consecutive to nanostructuration is not supposed to induce a favorable impact on adhesion.

Apart from the chemical and wettability characteristics of the surfaces, the topographic aspect of the surfaces is essential for bacterial adhesion. Topographically modified surfaces with nanopillars features have been studied. Indeed, Xu *et al*. nanostructured polyurethane urea (PUU) with two sizes of square arranged nanopillars: one with dimensions of 700 nm in length and 400 nm in diameter and interpillar distance, and a second one with dimensions of 650 nm in length and 500 nm in diameter and interpillar distance. Using staphylococci (*S*. *aureus* Newman and *S*. *epidermidis* RP62A) they demonstrated a decreased bacterial adhesion and an inhibition of biofilm development on these two surfaces [13]. Hochbaum *et al*. developed square organized pillars in epoxy resin whose dimensions were diameters of 300 nm, lengths of 2μm and interpillar distances ranging from 0.9 to 4 μm [65]. Adhesion of *Pseudomonas aeruginosa* PA14 was sensitive to the spacing between adjacent pillars. When this spacing was greater than the length of *P*. *aeruginosa*, adhesion was random. When it was between 1.2 and 1.5 μm (about the length of the bacterium), *P*. *aeruginosa* adhered along the plots (direction of the square side then the diagonal square). At 0.9 μm spacing, bacteria were perpendicular to the substrate and parallel to the axis of the posts. Recently, Jin *et al*. developed nanopicots in polyethylene terephthalate whose dimensions were a diameter of 250nm, a length of 1μm and a variable interpillar distance of 300, 450 and 650nm [66]. Bacterial adhesion (*S*. *aureus*, *Escherichia coli* and *Helicobacter pylori*) compared to the control surfaces was reduced for the surfaces with the two wider interpillar distance.

To explain the effect of nanostructured surfaces on bacterial adhesion, several hypotheses could be proposed. Some authors have hypothesized that surface textures with sub-bacterial dimensions could reduce the material surface area accessible to bacteria, resulting in a decreased probability of interaction with the material surface and a reduction in bacterial adhesion [13]. Nevertheless, it appears that one of the critical features involved in the anti-adhesion efficacy of a motif could be the interpillar distance, which has been confirmed by the study of Jin *et al*. [65–67]. The impact of the nanostructured surfaces could also be a mechanical one, the contact between the bacteria and the top of the pillars leading to a bacterial cell wall bending followed by rupture due to the relative rigidity of the cell wall [68,69]. The comparisons of our nanostructured surfaces with modified surfaces having an impact on bacterial adhesion can lead us to propose some explanations about the lack of impact of our ABS surfaces. The bacteria-accessible surface area for the nanostructured surfaces represents about 28% of the control ones, which is the same order of magnitude of the surfaces developed by Xu *et al*. [13]. The size of the diameter and the length of the ABS features developed are smaller than the dimensions of nanopicots made by Xu *et al*. and Jin *et al* [13,66]. The impact of the nanofeature diameter with sub-100nm size on bacterial adhesion have been studied for TiO_2_ nanotubes and researches lead to conflicting results [70,71]. Then, the interpillar distance dimensions of nanopillars prepared in this study are around 100 nm whereas effective anti-adhesive surfaces have interpillar distance around 500 nm [13,66]. The dimensions of nanopillars prepared in this study, especially interpillar distances, may not reduce sufficiently the bacteria-accessible surface area and therefore it could be a promising way to manufacture nanostructured surfaces with higher interpillar distances.

## Conclusion

This work has documented the fabrication of nanostructured ABS surfaces with AAO molds using several replication techniques. Among these techniques, the most pertinent one for a possible future industrial transposition was selected. The ABS nanofeatures developed are composed of hexagonally arranged nanopillars whose dimensions are about 73 nm in length, 56 nm in diameter and with an interpillar distance of 100 nm. More importantly, in this study, we focused on the critical factors of the fabrication which should be controlled in order to limit any variation of the nanofeatures in an industrial process. The reproducibility of the AAO mold fabrication was assessed and found to be correct. The analyses of the dimensions of the ABS nanofeatures showed low coefficients of variation for diameters and interpillar distances but higher coefficients for the length parameter. The wettability study has shown that the flat control ABS surface presents a slightly hydrophilic character. The nanostructuration of the polymer surface induces an increased apparent wetting, nervertheless, the contact angles study strongly suggests that this change in surface apparent wettability is mainly due to a roughness effect. The biofilm adhesion test on the ABS surfaces indicates that the nanostructuration has no effect on bacterial adhesion for *S*. *epidermidis*.

In conclusion, we have developed a strategy to produce and characterize polymeric nanostructured surfaces with AAO molds in an industrial perspective and to test their impact on microbial biofilms. This strategy could be implemented to test other materials such as polypropylene or silicone and other morphological features of the nanostructures such as a higher interpore distance.

## Supporting Information

S1 File Data for tables and Fig 5. (XLSX)

S2 File (XLSX)
